# Research on Land Use Planning Based on Multisource Remote Sensing Data

**DOI:** 10.1155/2022/5851768

**Published:** 2022-06-29

**Authors:** Wei Jia, Tingting Pei, Kai Lei

**Affiliations:** ^1^School of Food and Environment, Jinzhong College of Information, Jinzhong 030800, China; ^2^School of Architecture and Urban Planning, Lanzhou Jiaotong University, Lanzhou 730070, China

## Abstract

Land use changes are analyzed correctly, a series of improvements according to the changes are carried out appropriately, the relationship between land use development and economic and human survival is handled correctly, and the healthy and orderly development of the entire society is promoted. Aiming at the combination of multisource remote sensing data and monitoring changes in land planning, this study uses CBERS data and ASAR data as multisource remote sensing data sources to conduct in-depth research and discussion on the land use change in this area and uses the HPF pixel-level fusion method for data fusion to generate HPF. The data are integrated, and then, the CBERS data and HPF fusion data are used to extract the land use type information of Zhenning County, respectively, and a confusion matrix is built based on the field sample points to verify the accuracy, compare and analyze the relative error of the land use type information extraction before and after data fusion, and evaluate the CBERS data. Regarding the extraction effect of land use type information of fusion data with HPF, the results show that the two kinds of remote sensing data have good effects in extracting water body type information, and the accuracy has reached 100%. Using multisource remote sensing image processing can well summarize and analyze land use changes and make the changes in various indicators in the study area. Accurate statistics is obtained.

## 1. Introduction

To accelerate the pace of my country's economic construction, urbanization should be promoted. In the 2030s, my country should build a well-off society in an all-round way. The basic guarantee conditions for a moderately prosperous society are the construction of new towns, the rational use of limited land resources on the basis of existing rural land, and the completion of real estate construction and the construction of surrounding supporting facilities. The people-oriented concept to the stage of rural land planning is implemented, to achieve efficient use of existing land resources; in line with the basic concept of national construction at this stage, limited rural land resources must be planned and utilized in the early stage of construction, and to solve the problems that often occur in the land planning stage, people-oriented management concepts should be adopted to formulate and implement policies [1, 2]. People-oriented management is in line with the future development of contemporary construction and management. It is people-oriented, implements basic concepts in the process of land planning, adopts strict management methods to reduce the occurrence of corruption incidents, and promotes the construction of new rural and urban systems in my country [3].

The virtuous circle of land use system is determined by the rationality of land use structure. Land use changes are analyzed correctly, a series of improvements are carried out appropriately according to the changes, the relationship between land use development and economic and human survival is handled correctly, and the healthy and orderly development of the entire society is promoted [4, 5]. This study analyzes the dynamic change characteristics of its land use and obtains the changes in cultivated land, vegetation, roads, residential land, and water areas, as well as the trend of human factors [3, 6, 7].

As a long-distance, noncontact target detection technology and method, remote sensing has the characteristics of multiplatform, multiband, and multilevel and has the advantages of abundant information, short information acquisition period, and strong real-time and dynamic characteristics. It can detect the target, obtain information, after processing, locate, and qualitatively and quantitatively describe the target [8–12]. Land use type information can be obtained by applying remote sensing technology and related geoscientific analysis, and thematic map production can be performed, which makes remote sensing technology have incomparable advantages in the field of land use type classification. Therefore, the use of remote sensing data to extract land use type information is an important means of classifying land use types. At present, the use of remote sensing data to quantitatively extract land use information in the study area is more and more important in the field of environmental science research such as large-scale regional soil erosion [13]. However, in the classification of land use types, there are limitations in data sources and extraction methods. The main problems are that the amount of data is small, the data source is single, mostly optical remote sensing, and radar data are rarely used, the spectrum is not rich, and the resolution needs to be improved. Multisource remote sensing data fusion can enrich the remote sensing information of various sensors, and use the redundancy and complementarity of multisource remote sensing information to describe the target or scene information to the greatest extent [14–16]. CBERS optical remote sensing spectral information is rich, but the spatial resolution is low, and it is easily affected by weather, while ASAR data have high spatial resolution and are less affected and restricted by weather. Therefore, the fusion of CBERS and ASAR remote sensing data can make CBERS optical remote sensing data and ASAR radar remote sensing data form complementary advantages, thereby improving the ability of target recognition, analysis, and interpretation [17–19].

This study uses CBERS and ASAR data as multisource remote sensing data sources, uses HPF pixel-level fusion method for data fusion, then uses the data before and after fusion to extract land use type information in Zhenning County, and compares and analyzes the relative error of land use type information before and after data fusion, and compares the extraction effect of land use types before and after fusion. The research results have important guiding significance for the extraction of land use type information in Zhenning County, a typical karst area with serious soil erosion and fragile ecological environment. In addition, land use type, as an important evaluation factor of soil erosion, is also the main parameter for constructing the soil erosion model in the study area. Therefore, the research on the extraction of land use type information based on multisource remote sensing data can provide a reference for the extraction of land use type information in large-scale soil erosion information and is of great significance for the analysis and evaluation of soil loss in large-scale research areas.

## 2. Research Progress of Remote Sensing Classification Based on Multisource Data

The accuracy of remote sensing classification is usually affected by the spatial and temporal resolution of the data. In the process of land use classification and crop planting information extraction, remote sensing images with a certain spatial resolution and time series are often required to ensure accuracy. Currently, remote sensing data have products with different temporal, spatial, and spectral resolutions. However, usually high spatial resolution data, due to the long return visit period, insufficient description of the key phenological period of crops, insufficient temporal characteristics, and small width, limit the application of the data in a large range; the ability to describe the space is not high, and there are many mixed pixels. The spatial and temporal resolutions often restrict each other, and it is difficult for the existing remote sensing data to take into account both and cannot satisfy the higher temporal and spatial resolutions at the same time. Therefore, many experts and scholars have carried out research on multisource data fusion technology to complement data from different sources, obtain as much spatial information and temporal features as possible, and obtain data with high spatial and temporal resolution. At present, fusion technology is mainly divided into two categories according to different goals: multisource data fusion technology that improves spatial resolution and temporal resolution. Through data fusion technology, the improvement of spatial resolution and the increase in spatial information can reduce the influence of mixed pixels on the accuracy, but there will still be certain errors, which will cause distortion of spectral information to a certain extent; for the improvement of temporal resolution, obtaining more specific time-series changes makes the temporal characteristic differences in ground objects more significant and reduces errors caused by the intersection of short-term phenological periods [20].

Due to the complex topography and changeable topography types in the study area, the distribution of ground objects is affected and the spatial heterogeneity is large. In addition, the agricultural planting structure in my country is complex and diverse, the fields are fragmented, the mixed pixel phenomenon is serious, and the fragmentation of the fields and other factors will also affect the classification accuracy of remote sensing. The accurate identification of ground objects and the correct classification of land use types require high spatial resolution of remote sensing data [21–23]. On the one hand, a reasonable model is used to decompose the mixed pixels, which can improve the classification accuracy to a certain extent. Jia et al. believed that the temporal features extracted from low-resolution images are of great significance for improving the classification accuracy of high spatial resolution remote sensing images; in particular, for vegetation types, the classification results have higher accuracy.

Multisource data fusion technology generally uses a certain method to process and operate remote sensing data from different sensor sources and different spatial resolutions according to certain rules to obtain higher spatial resolution. This information includes spectral reflectance, backscattering coefficient, and time-series vegetation index. These methods are generally based on color correlation techniques, such as HIS transform method, or based on statistical methods, such as PCA, and Brovey and wavelet transform. The more extensive is in the data fusion of low spatial resolution and medium resolution with a certain time series.

Considering the texture features, spatial features, and other information of the study area is of great significance to further improve the accuracy of land use classification. The research shows that considering the spatial distribution characteristics and mutual correlation of ground objects can effectively make up for the shortcomings of pixel classification methods based on remote sensing images. Shao Wei et al. obtained the regional slope distribution law from the DEM statistical information of each category and used it as a classification rule in the classification postprocessing, which improved the problem of missing classification of some land categories. Jia et al. used the multitemporal HJ-1 satellite CCD data in 2010 as the main data source, supplemented by Landsat TM image, DEM, slope, and feature index data, and adopted the object-oriented classification method to extract the Guanzhong plain artificial surface information, higher classification accuracy. The object-based image analysis methods such as Jiang Fan comprehensively used the spectrum, texture, shape, slope, and other characteristics of image objects to realize the thematic mapping of land use/land cover in Qinwangchuan area in various periods. It improves the classification efficiency and increases the reliability of the classification results. In addition, with the development of time-series analysis [24–28], some researchers combine time-series analysis methods to analyze.

## 3. Land and Building Research Model Based on Multisource Data Fusion

### 3.1. Research Area

Zhenning County is located in the southwest of the hilly plains in the central part of Guizhou, at 105°35′10″–106°0′50″ east longitude and 25°25′19″–26°10′32″ north latitude. The county has a land area of 1713.3 km^2^, and the terrain is high in the north and low in the south, with a large slope variation, with an altitude of 356–1678 m. Zhenning County is a typical karst area, with a mountainous area of 1098 km^2^ and a hilly area of 157.8 km^2^, accounting for 64.08% and 9.21% of the county's total area, respectively. Karst landforms are widely distributed, accounting for more than 60% of the county's total area. The bedrock is mostly limestone, sand shale, dolomitic limestone, and siliceous limestone, and the soil is mostly yellow soil, red-yellow soil, and black lime soil.

### 3.2. Data Sources

CBERS data and ASAR data are taken as multisource remote sensing data sources. CBERS data come from the resource environment cloud platform, ASAR data come from ESA, and DEM data with 30 m resolution are selected as auxiliary classification data. CBERS images have a large number of spectrums and have the advantages of better definition and higher geometric accuracy, but they also have some disadvantages such as a certain degree of fringe dislocation and poor resistance to atmospheric interference. ASAR images have high spatial resolution, all-weather, penetrability to the surface and clouds, and diversity of information carriers.

### 3.3. Image Preprocessing

CBERS data adopt the software ENVI 5.0 software, first synthesize the first 4 bands, select UTM for image projection, select ground control points (GCPs), perform geometric correction and fusion on the 5th band, and then use Zhenning County vector data to align the CBERS data. The CBERS image is cropped, and finally, the cropped data are processed by image transformation and enhancement to generate the CBERS image of the study area. After the ASAR data are processed by speckle noise removal, image calibration, filtering, and geometric correction are carried out, the ASAR image of the study area is cropped, and the CBERS and ASAR remote sensing images within the study area are fused by the high-pass filter (HPF) fusion method, to generate HPF fusion data.

### 3.4. Classification of Land Use Types

In this study, the random forest classification method is used to classify the study area, and the sample and feature space of each type of ground object are defined as the reference basis for classification, and the classification is performed according to the statistical characteristics of the image itself and the distribution of natural point groups. According to the research object and the standard of the national land use classification system, the land use types are divided into 8 categories: grassland, buildings or rocks, mountains, sloping farmland, forest land, other forest land, water body, and open land.

The random forest (RF) algorithm is a nonlinear and nonparametric classifier proposed by Breiman et al. in 2001, which allows the fusion of high-dimensional data from multiple sources, and has a high tolerance for missing values and outliers. It is suitable for high-dimensional complex datasets and can automatically judge the importance of variables. It takes the decision tree as the basic unit, integrates several decision trees through the idea of ensemble learning, uses multiple trees to train and predict the samples, and uses voting to determine the classification results of the samples. By constructing different sample training sets, random forest expands the difference between the classification models of decision tree, thereby improving the extrapolation prediction ability of the combined classification model. After obtaining a classification model sequence {*h*_1_(*X*), *h*_2_(*X*),…, *h*_K_(*X*)} through K rounds of training, a multiclassification model system is formed at this time, and the final classification result is obtained by adopting a simple majority voting decision. The classification decision is as follows:

(1) $$\begin{array}{c} \text{H}\left(x\right)=\text{argmax}\sum_{i=1}^{K}I\left(h_{i}\left(x\right)=Y\right), \end{array}$$

where *H*(*x*) represents the combined classification model, *h*_*i*_ represents the single decision tree classification model, *Y* represents the output variable, and *I*(°) is the indicative function. In the process of constructing the random forest classification algorithm, it is necessary to set the number of decision trees and ensure the maximum number of features when the model is optimal. In this study, the grid search cross-validation method was used for parameter optimization. Through a large number of experiments, it is found that the error gradually converges and tends to be stable when it is set to 300. At this time, the maximum number of features is set to the square root of the total number of features *n*.

### 3.5. Classification Result Evaluation Method

In the accuracy evaluation, the sampling method is used to replace the whole image with some pixels or some categories to evaluate the accuracy of the image. Image accuracy refers to the degree of agreement between an image whose quality is unknown and a hypothetically accurate reference image or an image in the training area of a ground truth sample. An error matrix (or confusion matrix) is established to calculate various statistics and perform statistical tests and finally give the classification accuracy values for the overall and based on various ground types, error and misclassification error 4 indicators.

#### 3.5.1. Drawing Accuracy

The likelihood is that the classifier can assign a pixel in a picture to class A, assuming the ground truth is class A. It is used to reflect how good or bad the method of producing this graph is.

(2) $$\begin{array}{c} P_{AJ}=\frac{P_{A}\text{area}}{P_{J}\text{area}}, \end{array}$$

where *P*_*AJ*_ is the percentage, *P*_*A*_*area* is the PA area, and *P*_*J*_*area* is the PJ area.

#### 3.5.2. User Precision

Assuming that the classifier assigns cells to class A, the corresponding ground truth class is the likelihood of A. It reflects the credibility of each category in the classification map, that is, the reliability of the map.

(3) $$\begin{array}{c} P_{Ui}=\frac{P_{U}\text{area}}{P_{i}\text{area}}, \end{array}$$

where *P*_*Ui*_ is the percentage, *P*_*U*_*area* is the PU area, and *P*_*i*_*area* is the Pi area.

The mapping accuracy and user accuracy correspond to the missed classification error and misclassification error. The missed classification error shows how many of the actual objects of a certain type are wrongly classified into other categories, while the misclassification error shows how many of the objects classified as a certain type in the image should actually belong to other categories, the misclassification error is complementary to the mapping accuracy, and the misclassification error is complementary to the user's accuracy. 1 sampling point representing 8 land use types is selected on the CBERS image and HPF fusion image, respectively, a total of 16 sampling points, and each sampling point has an area of 667 m^2^; a record table for the field survey and verification content of the land use types of the sampling points are formulated; field investigation and verification of sampling points are carried out, land use type information data are recorded in detail, the data of sampling points are summarized and counted to establish an error matrix formed by four indicators of mapping accuracy, user accuracy, missed classification error, and wrong classification error. The results of land use type information extracted from HPF fusion images were analyzed and evaluated.

## 4. Results and Analysis

### 4.1. Extract Results

**Figure 1:**
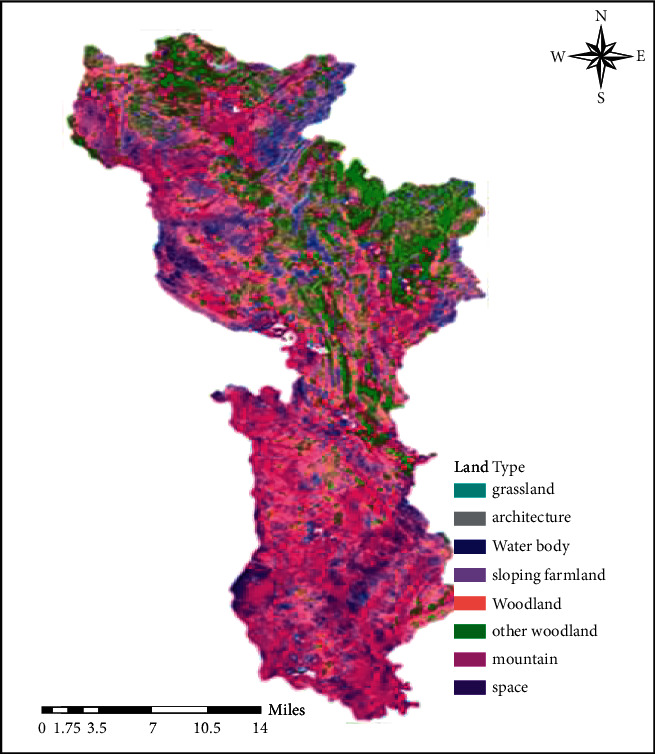
Classification of land use types in CBERS images.

**Figure 2:**
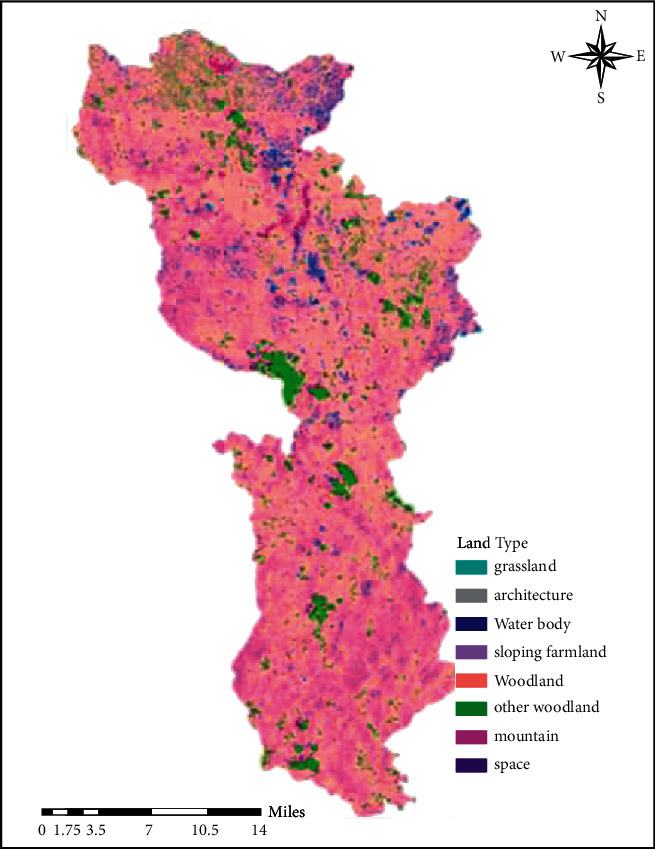
Classification of land use types of HPF fusion images.

The results of extracting land use type information based on CBERS images are shown in Figure 1, and the results of extracting land use type information based on HPF fusion images are shown in Figure 2.

### 4.2. Precision Analysis of Classification Results

The results and analysis of land use type information extraction are obtained based on CBERS images.  Table 1 provides the results of land use type information extracted based on CBERS images: except for grassland types and other forest land types, which have relatively low accuracy, other types have relatively high accuracy and good classification effects. The architectural or rock mapping accuracy is 93.6%, and the user accuracy is 96.9%; the water system mapping accuracy and user accuracy are both 100%; the slope farmland mapping accuracy is 91.9%, and the user accuracy is 88.8%; the forest land mapping accuracy is 91.6%, and the user accuracy is 91.6%. The accuracy is 87.5%; the mountain mapping accuracy is 95.7%, and the user accuracy is 100.0%; the air-ground mapping accuracy is 91.2%, and the user accuracy is 89.9%. From Table 1, it can be seen that the mapping accuracy of grassland types is relatively low, and the phenomenon of missing classification is relatively serious. There are many pixels that belong to grassland types that are divided into other woodlands, but the user accuracy is high, and the misclassification error is small. The main reason is the spectral characteristics of CBERS. In the image, the texture and tone of each land use type are more obvious and easy to distinguish; the user accuracy of other forest land is low, and the misclassification error reaches 15.2%. The image boundary between shrubland and tree forest is not obvious, which leads to misclassification.

The results and analysis of land use type information extraction are obtained based on HPF fusion images.  Table 2 provides the results of land use type information extracted based on HPF fusion images: each land use type has high mapping accuracy and user accuracy, and the missed classification error and misclassification error are small, and the classification effect is better. The water system mapping accuracy and user accuracy are both 100%; the grassland mapping accuracy is 96.5%, and the user accuracy is 98.8%; the building or rock mapping accuracy is 98.5%, and the user accuracy is 100%; the mountain system mapping accuracy is 97.1%, and the user accuracy is 97.1%; sloping farmland mapping accuracy is 96.3%, and user accuracy is 98.2%; forest land mapping accuracy is 98.2%, and user accuracy is 97.9%; other forest land mapping accuracy is 96.1%, and user accuracy is 96.3%; open land mapping accuracy is 96.1%, and the user accuracy is 92.1%; the mapping accuracy and user accuracy of all land use types have reached more than 96%. From Table 2, it can be seen that only the other two types of forest land and open land are prone to misclassification, and there is a misclassification error, but the errors are small, all less than 10%, indicating that the HPF fusion image extracts land use type information with high accuracy, which is more accurate than fusion. The higher image resolution is related to the clearer texture, tone, and boundary of the image patch of land use type.

### 4.3. Classification Result Accuracy Evaluation

In general, HPF fusion images are better than CBERS images in extracting land use type information. In terms of user accuracy, the three land use types of water body, building or rock, and mountain body extracted from HPF fusion images can reach 100% accuracy, while CBERS images only reach 100% for mountain types, indicating that the range of land use types extracted from HPF fusion images is wider. The user accuracy of HPF fusion images is above 90%, and there are not many misclassifications, indicating that the extraction effect of HPF fusion images is significantly better than that of CBERS images. In terms of mapping accuracy, HPF fusion images extract water and open space types, and the accuracy reaches 100%, while CBERS images can only reach 100% for water types; in addition to water types, the mapping accuracy of other land use types and HPF fusion image accuracy all are higher than CBERS images, indicating that the HPF fusion images have less missed classification and better results in extracting land use type information.

## 5. Conclusion

Aiming at the combination of multisource remote sensing data and monitoring the change in land planning, this study conducts an in-depth study and discussion on the land use change in this area. Using CBERS image and HPF fusion image as data sources, HPF remote sensing image fusion is carried out, and the random forest classification method is used to extract it. The land use type information of Zhenning County and the results are as follows:

1. In general, the extraction of land use type information based on HPF fusion images has fewer missed points, and the effect of extracting land use type information is better. In terms of extracting water body types, the accuracy of both CBERS image and HPF fusion image has reached 100%, and both have the same effect; for the extraction of other land use type information, the accuracy of HPF fusion image is higher than that of CBERS image, and the effect of HPF fusion data is better than that of CBERS image.
2. Using CBERS and ASAR images for HPF fusion, generating HPF fusion images, and extracting land use type information from HPF fusion images are one of the effective ways to improve the accuracy of land use type classification

## Figures and Tables

**Table 1:** Error matrix of land use type information extraction results based on CBERS image and HPF fusion image.

Land use type		Field survey area (m^2^)								
		Grassland	Building or rock	Mountain	Sloping farmland	Woodland	Other woodland	Water body	Space	Sum
CBERS image	Grassland	564	0	0	0	0	13	0	6	583
	Building or rock	7	638	0	0	0	0	16	12	659
	Mountain	10	0	651	7	0	0	0	0	670
	Sloping farmland	23	0	14	612	0	0	0	48	692
	Woodland	26	0	0	0	610	540	0	0	690
	Other woodland	36	0	0	15	58	113	0	0	714
	Water body	0	0	0	0	0	0	652	0	652
	Space	0	30	3	34	0	0	0	608	675
	Sum	668	668	668	668	668	668	668	668	5344

HPF fusion image	Grassland	642	0	0	1	15	0	1	0	659
	Building or rock	0	652	20	0	0	0	0	0	672
	Mountain	0	0	648	3	0	0	0	0	651
	Sloping farmland	0	16	0	663	0	0	0	0	679
	Woodland	0	0	0	0	638	0	0	0	638
	Other woodland	12	0	0	1	15	667	0	1	696
	Water body	14	0	0	0	0	0	667	0	681
	Space	0	0	0	0	0	1	0	667	668
	Sum	668	668	668	668	668	668	668	668	5344

**Table 2:** Accuracy evaluation of land use type information extraction results based on CBERS images and HPF fusion images.

Land use type	CBERS image				HPF fusion image			
	Drawing accuracy (%)	Drawing accuracy (%)	User accuracy (%)	Misclassification error (%)	Drawing accuracy (%)	Drawing accuracy (%)	User accuracy (%)	Misclassification error (%)
Grassland	84.5	14.9	97.1	3.6	96.5	3.4	98.8	1.3
Building or rock	93.6	5.8	96.9	3.2	98.5	1.2	100.0	0.0
Mountain	95.7	3.5	100.0	0.0	97.1	2.3	100.0	0.0
Sloping farmland	91.9	8.1	88.8	11.5	96.3	3.1	98.2	1.2
Woodland	91.6	8.4	87.5	12.3	98.2	1.6	97.9	2.0
Other woodland	89.9	10.5	84.1	15.2	96.1	3.5	96.3	3.2
Water body	100.0	0.0	97.6	2.5	100.0	0.0	100.0	0.0
Space	91.2	9.8	89.9	10.8	100.0	0.0	92.1	7.3

## Data Availability

The data used to support this study are available from the corresponding author upon request.
